# Accidental Stent Extraction Following a Rescue Manipulation to Retrieve a Lost Coronary Guidewire: A Very Rare Complication

**DOI:** 10.1002/ccr3.70612

**Published:** 2025-07-08

**Authors:** Réza Dadkhah, Mihai Ciocoi, Elias Bentakhou, Claudiu Ungureanu

**Affiliations:** ^1^ Division of Cardiology Tivoli University Hospital La Louviere Belgium; ^2^ Cardiovascular Department Institutul Inimii Cluj Romania; ^3^ Department of Cardiology CHU Helora Jolimont Hospital La Louviere Belgium

**Keywords:** coronary stent retrieval, intravascular coronary imaging, percutaneous complex coronary interventions, severe calcified coronary disease

## Abstract

We present a complex angioplasty case in which a coronary guidewire became trapped between a calcified nodule and a newly implanted stent, leading to wire rupture and subsequent accidental stent extraction. This case underscores the importance of intracoronary imaging and highlights the potential procedural challenges and rare complications in modern percutaneous coronary intervention (PCI).

## 1 Introduction

Severely calcified coronary lesions remain a major challenge in contemporary interventional cardiology. According to recent meta‐analyses, such lesions are encountered in up to 24% of percutaneous coronary interventions (PCI) [1]. The prevalence of calcified lesions is expected to increase due to aging populations and the rising burden of comorbidities, such as diabetes, and chronic kidney disease [2]. Severe coronary calcification is an independent predictor of major adverse cardiovascular events post‐PCI [3]. Additionally, it increases procedural complexity and the risk of complications, including coronary dissection, perforation, balloon rupture, and suboptimal stent expansion [4].

This report presents a complex case illustrating the challenges associated with treating calcified coronary lesions. The case involves wire entrapment leading to its fracture and an exceedingly rare event, a stent extraction. Despite employing advanced plaque modification techniques, such as lithotripsy and rotational atherectomy, stent expansion remained inadequate. The use of intravascular imaging (IVI) was instrumental in evaluating plaque morphology, guiding lesion preparation, and ultimately optimizing the final procedural outcome.

## 2 Case History

**FIGURE 1:**
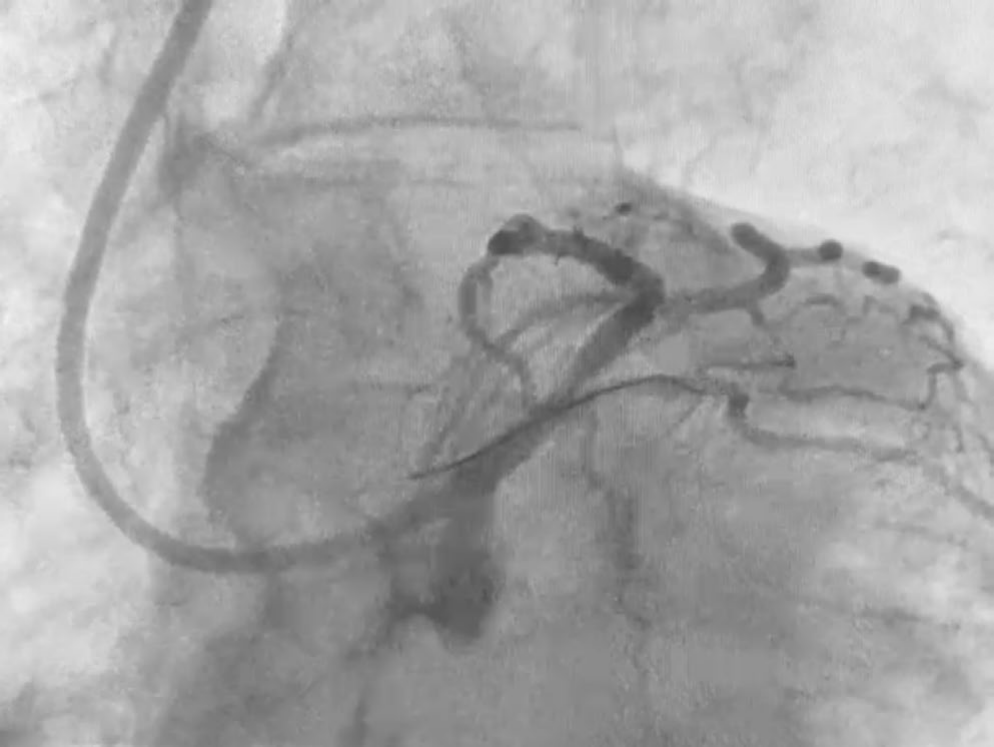
Angiography revealing the broken guide wire positioned into the circumflex artery and floating inside the aorta, side to the guiding catheter.

A 69‐year‐old man with a history of hypertension and hypercholesterolemia presented with unstable angina. Coronary angiography revealed severe stenosis of the right coronary artery (RCA), which was successfully treated with percutaneous coronary intervention (PCI). However, a complex ostial lesion of the left anterior descending (LAD) artery involving the left main coronary artery (LMCA) was also identified, necessitating a staged procedure. The patient was pretreated with aspirin (100 mg daily) clopidogrel (75 mg daily). A 6F transradial approach was used, with a 6F extra‐backup guiding catheter (EBU) positioned in the LMCA and two 0.014‐in. Sion blue wires placed into the LAD and Circumflex (Cx) arteries. Given the severity of calcification, lesion preparation included non‐compliant balloon dilatation and intracoronary lithotripsy. A 3.5 × 12 mm Shockwave balloon delivered 80 pulses to the lesion, followed by additional non‐compliant balloon dilatations. A 3.5 × 18 mm Xience drug‐eluting stent (DES) was deployed, covering the LMCA and proximal LAD. Proximal optimization technique (POT) was performed with a 4 × 8 mm non‐compliant balloon inflated to 20 atm. Upon attempting to reposition the Cx wire, significant resistance was encountered, suggesting wire entrapment between the stent and the calcified lesion. Unfortunately, attempts to retrieve the wire led to its fracture, with the distal broken segment trapped by the stent, and the proximal portion floating in the aorta (Figure 1). Several retrieval techniques were unsuccessful, including snaring and bioptome use, prompting the decision to halt the procedure. Although surgical retrieval was considered, it was deemed unnecessary due to the patient's hemodynamic stability.

**FIGURE 2:**
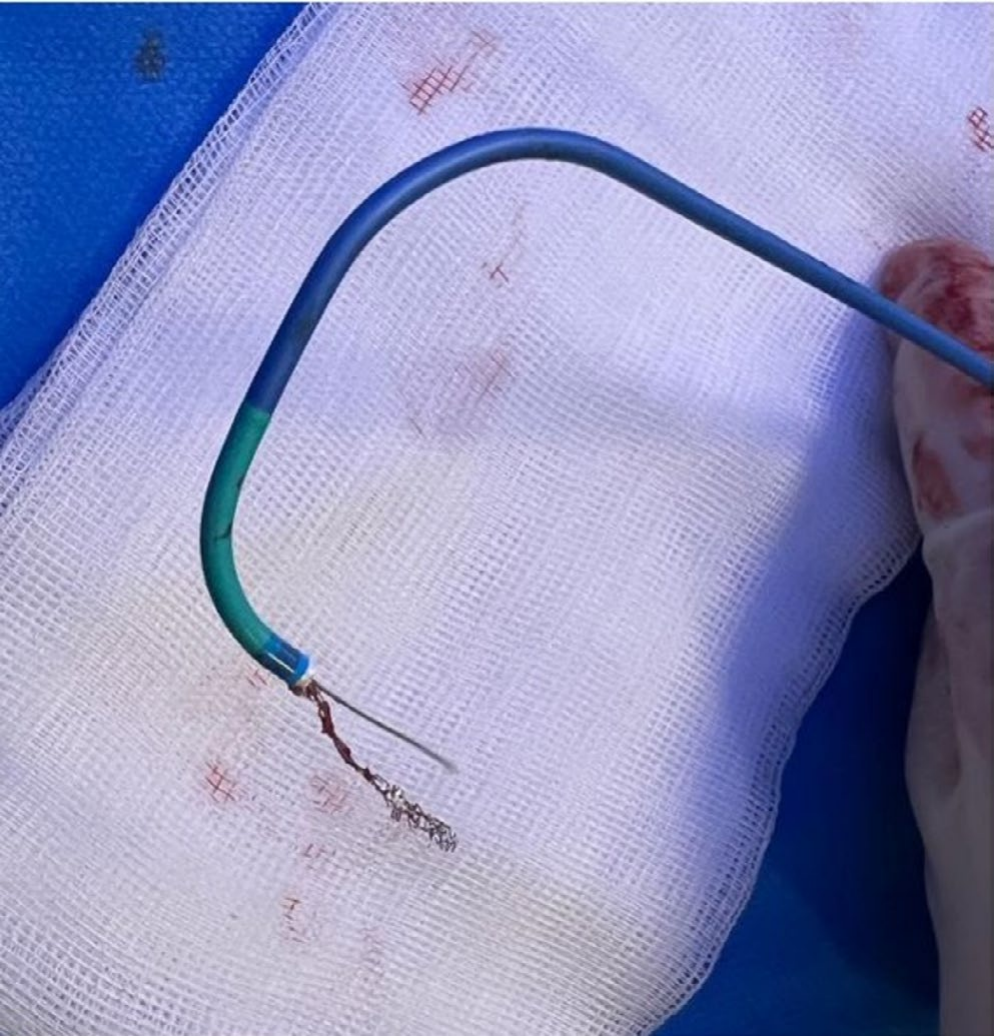
Ruptured wire out of the guiding catheter, next to the extracted stent (under the wire).

**FIGURE 3:**
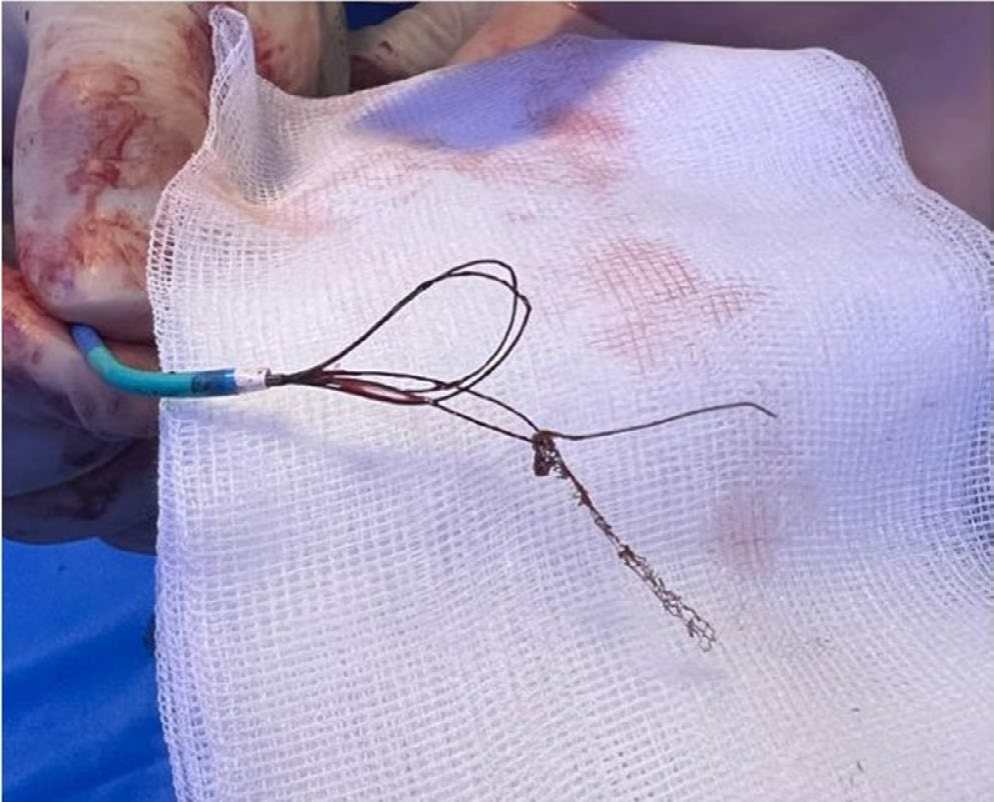
Multiple snare loop out of the guiding catheter, capturing both the fractured guide wire and the implanted stent.

**FIGURE 4:**
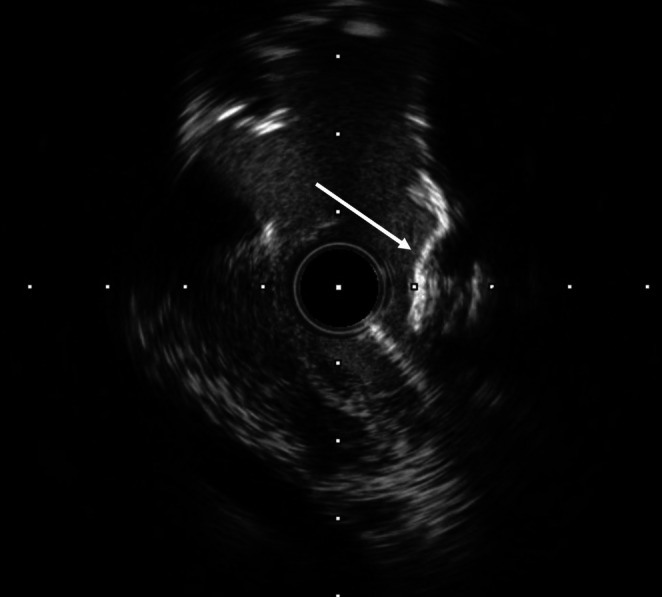
IVUS image revealing a heavily calcified nodule with an irregular, sharp‐angled, toothed structure, forming a cage‐like environment that trapped the wire after stent implantation.

A second elective procedure was performed 1 month later, using a 7F guiding catheter. After multiple failed attempts with a single snare loop, a multiple snare loop technique was successfully used to extract the fractured guidewire. However, this maneuver unexpectedly led to the complete extraction of the previously implanted stent, an extremely rare occurrence likely resulting from entrapment of the wire within the stent struts and extensive calcified plaque modification (Figures 2 and 3). Subsequent IVUS imaging revealed a severely calcified nodule at the ostium and a thick calcified arc in the proximal LAD (Figure 4). Despite previous coronary lithotripsy, the lesion remained incompletely expanded. Rotational atherectomy with a 2.0 mm burr was performed, followed by a second Shockwave balloon inflation, delivering 120 pulses. However, optimal plaque modification was only achieved with a 4 × 15 mm Wolverine cutting balloon, which created multiple deep fractures, allowing for the successful implantation of a 4 × 18 mm Xience DES. The procedure concluded with classic POT and final kissing balloon inflation achieving a satisfactory result.

## 3 Differential Diagnostic

When faced with the difficulty of an immobilized guidewire that cannot be removed, it is essential to consider various differential diagnoses to optimally manage this complication. One must consider the possibility of entanglement with another guidewire in place, which would shift efforts toward addressing the first guidewire. A blockage caused by the recently implanted stent should also be considered, prompting the use of small‐diameter balloons or microcatheters to free the guidewire. Furthermore, in this context of a wire losing mobility, it is advisable to avoid performing POT (proximal optimization technique), as it could worsen the situation. Finally, a conflict caused by vessel calcifications or severe tortuosities is another possibility to consider, which could necessitate alternative strategies, such as selecting different guidewires.

## 4 Outcome and Follow‐Up

The guidewire fracture in this context of significant coronary calcifications was resolved through this exceptional stent extraction, but it shifted our strategy toward better preparation of the calcified plaque, particularly through the use of intracoronary imaging. The angioplasty outcome was ultimately excellent, with no negative consequences from these procedural complications. The patient was seen again 6 weeks after this particularly challenging angioplasty, with no clinical complaints and, moreover, a complete resolution of angina symptoms.

## 5 Discussion

**FIGURE 5:**
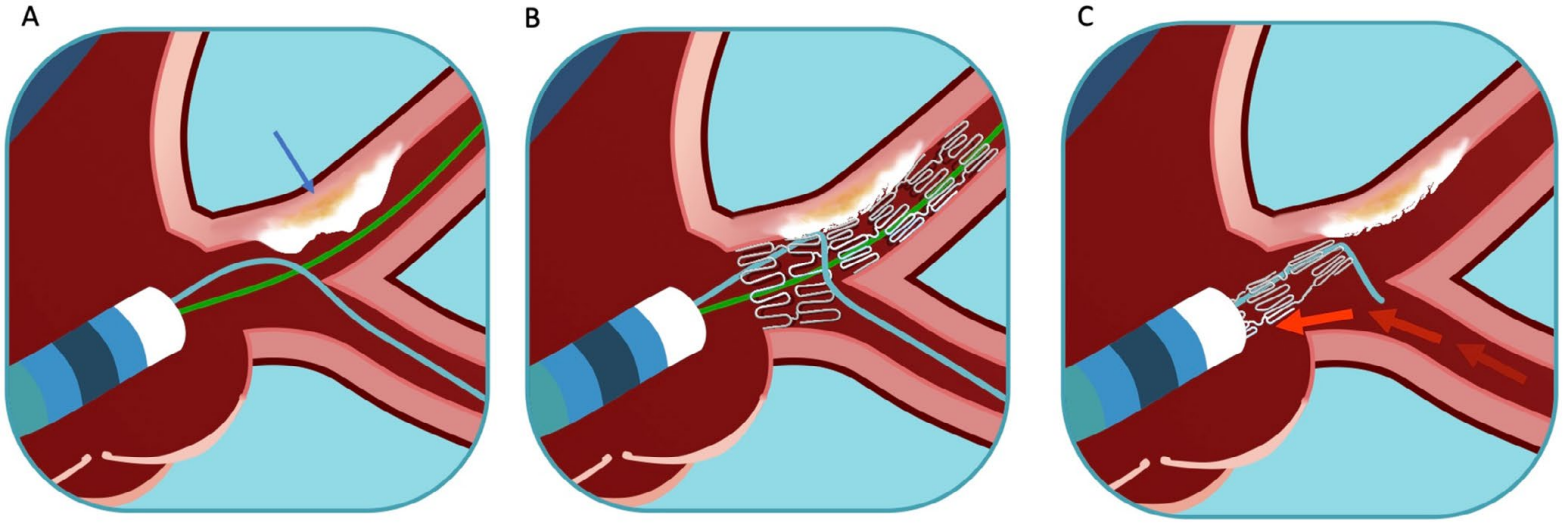
The stent extraction complication. (A) Treatment of the LMCA with 2 wires positioned, respectively in the IVA and Cx arteries. A severe calcium plaque (arrow) is visualized at the end of the LMCA. (B) The implanted DES is covering the LMCA and the prox IVA, with an enlargement of the proximal section obtained by POT. The wire in the Cx (light blue) is trapped by the stent and by the calcified plaque. (C) The maneuvers applied to retrieve the wire trapped in the Cx will lead to the stent extraction.

Guidewire entrapment and fracture is a rare but serious complication of PCI occurring in approximately 0.1%–0.2% of cases [5]. This risk is significantly heightened in the presence of severe calcifications, tortuous anatomy, and bifurcation lesions with jailed wires. In our case, the Cx wire was trapped between the newly implanted stent and an extensive calcified plaque; entrapment was probably worsened by the POT maneuver, ultimately leading to wire rupture and an unanticipated stent extraction (Figure 5).

Possible complications of fractured wires include acute vessel occlusion, coronary perforation, and distal embolization [6]. Conversely, some cases in the literature describe a non‐aggressive approach where the retained wire segment is treated conservatively with no clinical sequelae on long‐term follow‐up. Similarly, small fractured wires can be left within a chronically occluded coronary artery without adverse effects [7]. The decision to retrieve a wire depends on the specific situation and must be evaluated individually. If retrieval is necessary to avoid surgical intervention, a thorough understanding of various percutaneous techniques is crucial.

We have already addressed the use of bioptome, as well as the application of both single and multiple snare loops. Other techniques include the double or triple wire technique, which involves passing additional wires alongside the entrapped wire, then torquing all wires in a twisting motion to wrap them around the trapped wire. This twisted group of wires can then be retracted, pulling the entrapped wire out of the coronary artery toward the guiding catheter [8]. For wires floating in the aorta, the balloon inflation technique can be effective: the lost wire is caught inside the guiding catheter, a balloon is advanced and inflated at the distal part of the guiding catheter, tightly trapping the broken wire, which is then retracted [9]. If the wire is trapped but not fractured, using small balloons or a microcatheter can help dislodge it. This technique can also prevent wire fracture and safely retrieve the wire. Depending on the position and length of the lost wire and the coronary configuration, another option is to deploy a stent to secure the wire against the vessel wall. However, these percutaneous manipulations can lead to serious complications, such as dissection, perforation, or stent thrombosis.

In our particular case, the proximal part of the fractured wire was floating in the ascending aorta, posing a potential risk for thrombus formation and subsequent systemic embolization. During the wire retrieval by snaring, the complete stent frame was also unexpectedly extracted. A similar case was published by Varvarovsky and Matejka in 2007, in which the treatment of a bifurcation lesion in the circumflex artery was complicated by a jailed wire trapped in the obtuse marginal branch [10]. After retrieving the entire system, including the guiding catheter and the wires, the freshly implanted stent was also extracted, possibly due to the short length of the stent and inadequate anchoring in the proximal main branch. More recently, Kumar et al. published a paper on coronary wire entrapment leading to the extraction of a deployed stent. In this case, the newly implanted stent was deformed due to the traction on the jailed wire, and the operator had to extract the damaged stent together with the entrapped wire by pulling an inflated balloon positioned inside the stent, facilitating the removal of the material [11].

The peculiarity of our case lies in the “wrapping” interaction between the curvilinear stent struts, the jailed wire, and the non‐polished, unfractured calcium nodule. Additionally, the blazed‐shaped calcifications played a pivotal role in wire entrapment, increasing friction and resistance during retrieval attempts. This rare morphological configuration has not been widely documented in similar cases and underscores the complexity of treating severely calcified lesions.

Furthermore, to ensure optimal stent deployment, it is crucial to recognize that merely creating a single superficial fissure in a calcified plaque is often insufficient. The concept of “plaque decimation” [12] emphasizes the continuous fragmentation of atherosclerotic plaque through the use of dedicated devices or techniques, which is mandatory in cases of severe calcifications. Adequate plaque preparation not only enhances vessel compliance but also reduces the risk of mechanical damage to the stent structure or guidewire body, preventing adverse procedural outcomes such as what occurred in this case. These approaches ensure that the plaque is thoroughly fractured and softened before stent deployment, significantly reducing the likelihood of complications related to wire entrapment and suboptimal stent expansion.

During our second procedure, intravascular imaging (IVI) was instrumental in precisely detecting the anatomy and morphological aspects of the calcified plaque. Without IVI, many procedural nuances, such as the extent of calcium modification or the presence of deep fractures, would remain undetected, leading to suboptimal results and increased risk of complications.

In retrospect, a more aggressive plaque decimation strategy in the initial procedure, guided by IVI, might have prevented the wire entrapment and subsequent complications.

This case highlights the necessity of meticulous lesion assessment and preparation before stent implantation, particularly in the setting of complex calcified bifurcation lesions. IVI should be systematically used to evaluate calcium morphology, assess the effectiveness of plaque modification techniques, and ensure complete stent expansion, ultimately improving outcomes and long‐term patient prognosis.

To conclude, we have documented a highly uncommon complication that combined several pitfalls, rarely described in this sequence, where a jailed coronary wire, constrained by deformed stent struts over an extensively calcified nodular lesion, led to the complete extraction of the stent frame. This occurred during an attempt to snare the proximal fragment of a fractured wire. This case underscores the critical need for thorough plaque modification, which should be guided by intracoronary intravascular imaging, to avert such complications.

## Author Contributions

**Réza Dadkhah:** writing – original draft. **Claudiu Ungureanu:** writing – review and editing. **Mihai Ciocoi:** resources. **Elias Bentakhou:** resources.

## Ethics Statement

The authors are accountable for all aspects of the work in ensuring that questions related to the accuracy or integrity of any part of the work are appropriately investigated and resolved. All procedures performed in this report were in accordance with the ethical standards of the institutional ethical committee.

## Consent

Written informed consent was obtained from the patient for the publication of this case report and accompanying images. A copy of the written consent is available for review by the editorial office of this journal.

## Conflicts of Interest

The authors declare no conflicts of interest.

## Data Availability

The data underlying this article are available in the article.
